# Heat shock protein 90α reduces CD8^+^ T cell exhaustion in acute lung injury induced by lipopolysaccharide

**DOI:** 10.1038/s41420-024-02046-8

**Published:** 2024-06-13

**Authors:** Lei Yan, Yumei Chen, Yilin Yang, Yi Han, Chaoyang Tong

**Affiliations:** Department of Emergency Medicine, Zhongshan Hospital, Fudan University, 200032 Shanghai, China

**Keywords:** Respiratory distress syndrome, Immune cell death

## Abstract

CD8^+^ T-cell exhaustion is a promising prognostic indicator of sepsis-induced acute respiratory distress syndrome (ARDS). Patients with sepsis-related ARDS had reduced levels of HSP90AA1. However, whether the changes in CD8^+^ T cells were related to HSP90α, encoded by the HSP90AA1 gene, was unclear. This study aimed to examine the regulatory mechanism of HSP90α and its impact on CD8^+^ T-cell exhaustion in lipopolysaccharide (LPS)-induced acute lung injury (ALI). In this study, by conducting a mouse model of ALI, we found that one week after LPS-induced ALI, CD8^+^ T cells showed exhaustion characteristics. At this time, proliferation and cytokine release in CD8^+^ T cells were reduced. The inhibitory costimulatory factors PD-1 and Tim-3, on the other hand, were enhanced. Meanwhile, the expression of HSP90α and STAT1 decreased significantly. The in vitro studies showed that HSP90α stimulation or inhibition affected the CD8^+^ T-cell exhaustion phenotype. Interference with STAT1 reduced the expression of HSP90α and impaired its regulation of CD8^+^ T cells. The Co-Immunoprecipitation results indicated that HSP90α can directly or indirectly bind to TOX to regulate TOX expression and downstream signal transduction. In summary, by inhibiting TOX-mediated exhaustion signaling pathways, HSP90α inhibited CD8^+^ T-cell exhaustion in ALI. The participation of STAT1 in the regulation of HSP90α was required.

## Introduction

Sepsis is acute organ dysfunction caused by microbial invasion that disrupts the immunological response [1, 2]. The sepsis cytokine storm causes an exaggerated inflammatory storm, which often results in acute lung injury (ALI) and acute respiratory distress syndrome (ARDS) [3, 4]. More than 3 million individuals globally are diagnosed with ARDS each year, accounting for 10% of hospital critical care unit admissions [5]. Despite advances in medical technology, such as broad-spectrum antibiotics, mechanical ventilation, continuous renal replacement, and extracorporeal membrane lung oxygenation, ARDS patient mortality remains significant at ~40% [6].

CD8^+^ T cells are important for protective immunity against intracellular pathogens. Excessive amounts of antigen and/or inflammatory signals often lead to the gradual deterioration of CD8^+^ T-cell function, a state called “exhaustion”. In our previous study, we discovered CD8^+^ T-cell exhaustion in the blood of patients with ARDS [7, 8]. The number of CD8^+^ T cells and coinhibitory receptors (PD-1 and Tim-3) were promising independent prognostic markers of sepsis-induced ALI [8]. Transcriptome analysis results suggested that the HSP90AA1 gene was underexpressed in sepsis-associated ARDS [7]. However, it was unclear whether the changes in CD8^+^ T cells were related to HSP90α, which was encoded by the HSP90AA1.

HSP90α is a widely expressed and conserved molecule located in the cytoplasm, mitochondria, and endoplasmic reticulum of eukaryotic cells [9, 10]. It mediates the maturation and stabilization of intracellular proteins, facilitates the folding and remodeling of a variety of proteins, and is a key regulatory factor that affects cell growth, differentiation, and the apoptosis pathway [9, 11]. Hundreds of proteins depend on HSP90α chaperone activity, including kinases and transcription factors, and so HSP90α plays an important role in inflammation and cell growth [12–15]. Therefore, we hypothesized that HSP90α was involved in exhaustion and functional change in CD8^+^ T cells in sepsis-related ARDS.

In this study, we used a lipopolysaccharide (LPS)-induced ALI mouse model to observe the changes in phenotype and function of CD8^+^ T cells. We investigated the effect of HSP90α on CD8^+^ T-cell exhaustion. At the same time, we explored the interaction and possible regulatory mechanism of the HSP90α/STAT1/TOX pathway during CD8^+^ T-cell exhaustion. This study helps to clarify the exhaustion transition and potential mechanisms in CD8^+^ T cells with the progression of lung injury. Understanding the mechanism of CD8^+^ T-cell exhaustion is expected to provide new ideas for preventing and treating sepsis-related ARDS.

## Results

### Inhibition of HSP90α exacerbated LPS-induced lung injury

**Fig. 1:**
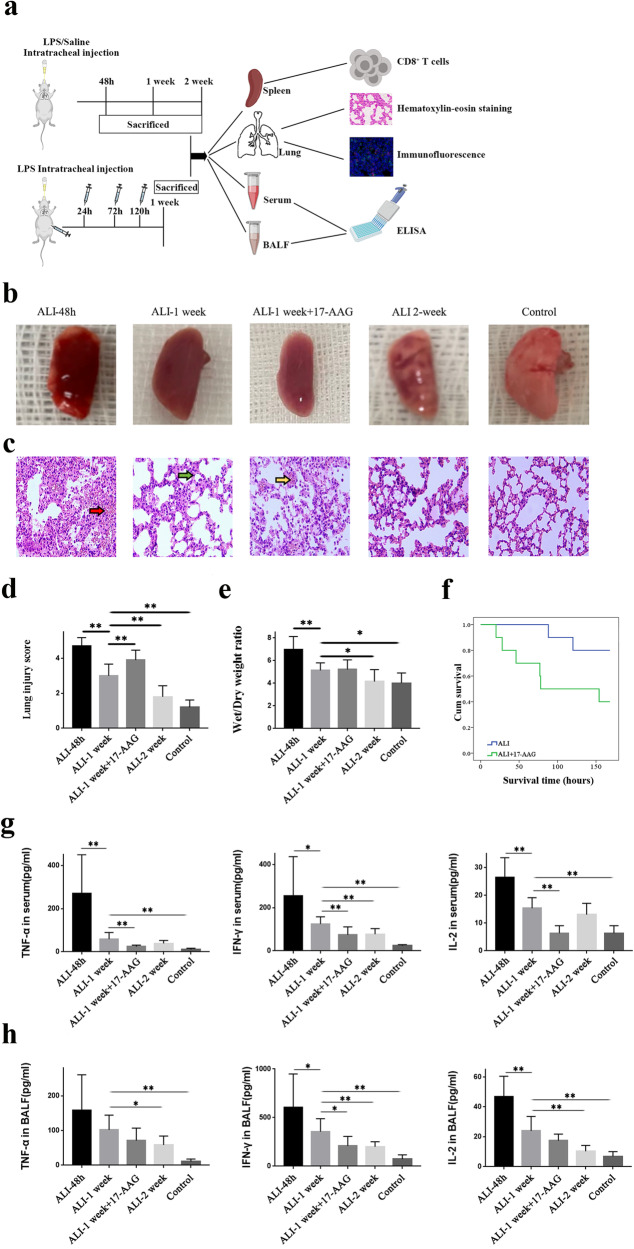
Changes in lung and systemic inflammation after ALI. **a** Schematic illustration of the experiments carried out in the ALI/17-AAG intervention groups. **b** Changes in gross lung tissues in mice treated with LPS for different times (48 h, 1 week, 2 weeks), the 17-AAG group, and the control group. **c** Microscopic lung tissue changes after HE staining. Inflammatory infiltration in lung tissue, alveolar epithelial hyperplasia, and local tissue bleeding are shown in red, green, and yellow. **d** Shows the statistical results obtained by the HE staining score in each group. **e** Statistical analysis of the ratio of wet weight to dry weight of lung tissue in each group. **f** The survival rate of mice injected with 17-AAG was lower than that of untreated ALI mice (*P* < 0.05). **g** and **h** Cytokine expression in serum and alveolar lavage fluid at different time points after ALI. *P < 0.05, **P < 0.01.

A sepsis-related ALI model was established by endotracheal infusion of LPS. The results of gross lung tissue specimens and HE staining are shown in Fig. 1b and c. In the control group, the appearance of the lung lobes was normal, and there was no obvious swelling or hemorrhage. Under a light microscope, HE staining showed that the trachea and alveolar structures were complete, the interstitium of the lung was evenly distributed, and no obvious bleeding or inflammatory cell accumulation was observed in the alveoli or interstitium. However, significant swelling, exudation, and diffuse bleeding were observed in gross lung tissue samples 48 h after ALI modeling. HE staining showed that the normal structure of alveolar tissue was destroyed at this point, accompanied by diffuse capillary hemorrhage and massive inflammatory cell infiltration in the alveoli and interstitial spaces of the lungs. In ALI mice without any intervention, the mice that were still alive after 1 week had reduced swelling in the lung tissue, less exudation, and less bleeding. There was less destruction of the alveolar wall as shown by HE staining, inflammatory cell infiltration in lung tissue was reduced, and intra-alveolar and interstitial hemorrhage was reduced compared with those at 48 h. Swelling and hemorrhage of the lung tissues were significantly reduced in mice that were still alive after two weeks. Additionally, inflammatory cell infiltration in lung tissues was further reduced, as shown by HE staining, hemorrhage in the alveoli and interstitium was absorbed, and the alveolar structures were gradually restored.

In the HSP90α inhibition group, we performed intraperitoneal injections of 17-AAG after LPS-induced ALI. After one week, we observed that lung tissue swelling appeared more obvious in the HSP90α-inhibited group than in the untreated group. Although the number of inflammatory cells in the lung tissue was reduced, the damage to the alveolar structure, alveolar exudation, and local bleeding remained severe, and the lung tissue injury score was higher than that of untreated ALI mice (Fig. 1b–e).

### Inhibition of HSP90α reduced inflammatory responses after ALI

IFN-γ, TNF-α, and IL-2 in serum and alveolar lavage fluid were detected at different time points by ELISA to examine the systemic and intrapulmonary inflammatory responses after ALI. As shown in Fig. 1g, h, the inflammatory response in blood and alveolar lavage fluid peaked 48 h after LPS injection, as shown by the production of IFN-γ, TNF-α, and IL-2. These cytokines decreased after 1 week but were still significantly higher than those in the control group. IFN-γ, TNF-α, and IL-2 levels in alveolar lavage fluid and TNF-α levels in serum declined after two weeks, and IL-2 in alveolar lavage fluid and TNF-α in serum approached control values. Compared with those of untreated ALI mice, the expression of IFN-γ, TNF-α, and IL-2 in serum and IFN-γ in alveolar lavage fluid was further decreased in the 17-AAG group, suggesting that the inhibition of HSP90α expression in vivo may weaken systemic and lung inflammatory responses.

### Inhibiting HSP90α reduced CD8^+^ T-cell infiltration during ALI

**Fig. 2:**
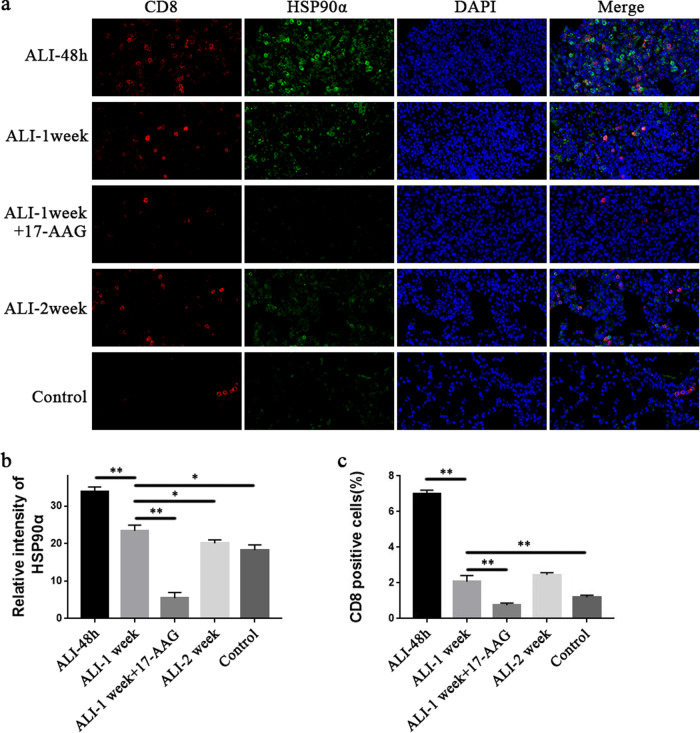
Double-labeled immunofluorescence analysis of CD8/HSP90α in lung tissue. HSP90α is shown in green, CD8 is in red, and the nucleus is in blue. **a** Representative immunofluorescence staining of CD8/HSP90α in lung tissue in the 48 h, 1 week, 1 week + 17-AAG, 2 weeks, and control groups after ALI induction. **b** Quantitative analysis of the mean fluorescence intensity of HSP90α in the lungs in each group of mice. **c** is the statistical result of the percentage of CD8-positive cells in the lungs in each group of mice. *P < 0.05, **P < 0.01.

The lung tissues of mice in each group were stained for CD8 and HSP90α (Fig. 2). Inflammatory cells, including CD8^+^ T cells, rapidly increased in lung tissue at 48 h after modeling. One week later, inflammation was decreased, and the infiltration of CD8^+^ T cells also decreased. HSP90α was highly expressed in inflammatory cells, including CD8^+^ T cells, in lung tissues at 48 h after modeling. Compared to that at 48 h, the expression of HSP90α was dramatically downregulated in lung tissue after 1 week and further decreased after 2 weeks. HSP90α was scarcely expressed in the lung tissues of mice in the 17-AAG intervention group, and CD8^+^ T cells in this group were also dramatically decreased (Fig. 2).

### Inhibition of HSP90α weakened the proliferation of CD8^+^ T cells

**Fig. 3:**
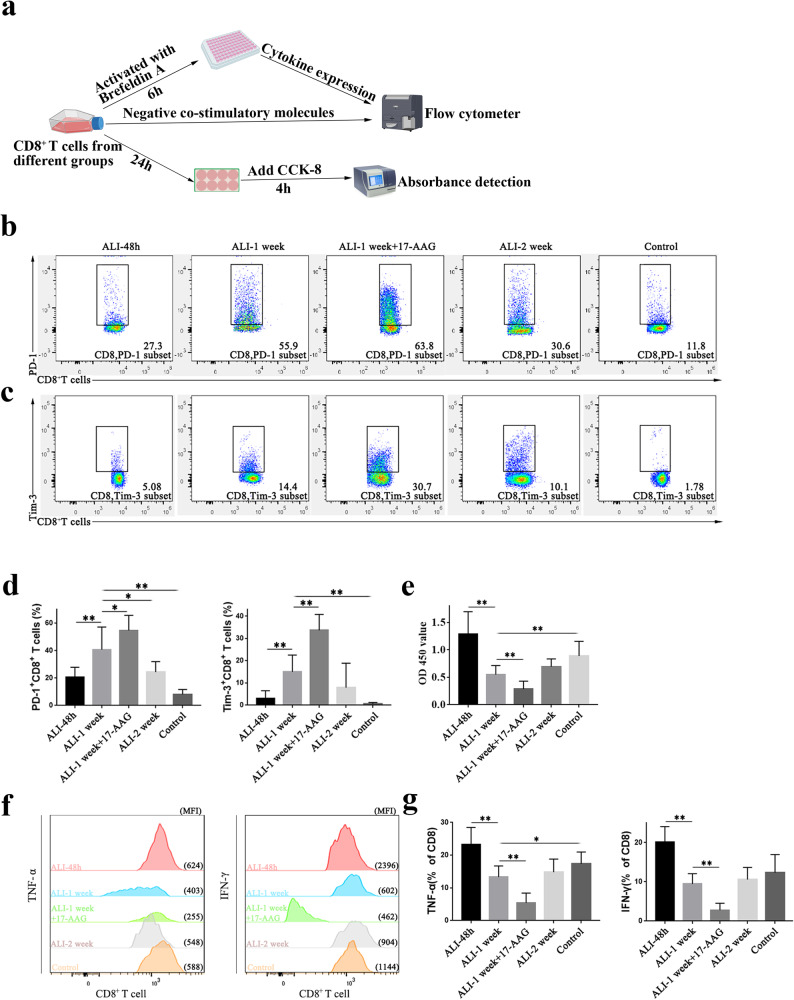
CD8^+^ T-cell exhaustion after ALI. **a** Schematic illustration of the experiments carried out on CD8^+^ T cells isolated from the ALI/17-AAG intervention groups. **b** and **c** Typical flow patterns of PD-1 and Tim-3 in CD8^+^ T cells from mice at 48 h, 1 week, and 2 weeks following intratracheal injection of LPS, as well as in the 17-AAG-treated and control groups. **d** The statistical analysis of the expression of the two negative costimulatory molecules. **e** The proliferation of CD8^+^ T cells in each group of mice. **f** Representative flow cytograms showing the expression of TNF-α and IFN-γ in CD8^+^ T cells in each group. **g** Statistical analysis of TNF-α and IFN-γ in CD8^+^ T cells in each group. *P < 0.05, **P < 0.01.

In this study, CD8^+^ T cells from each group of mice were isolated and cultured in vitro to examine their proliferative activity (Fig. 3e). These results suggested that CD8^+^ T-cell proliferation was most active in the acute phase of LPS-induced ALI (48 h) but decreased after 1 week. By 2 weeks after modeling, the proliferative capacity of murine CD8^+^ T cells was partially restored, but the OD values were not significantly different compared to those at 1 week. However, one week after intraperitoneal injection of 17-AAG, the proliferative activity of CD8^+^ T cells was worse than that in the untreated ALI group.

### Inhibition of HSP90α promoted CD8^+^ T-cell exhaustion

The expression of negative regulatory molecules (PD-1, Tim-3) in murine CD8^+^ T cells was analyzed by flow cytometry. As shown in Fig. 3b and c, in response to continuous inflammatory stimulation, CD8^+^ T cells overexpressed the negative costimulatory molecules PD-1 and Tim-3 after one week of ALI. However, after 2 weeks of modeling, the expression of exhaustion molecules in CD8^+^ T cells decreased with the alleviation of inflammation. The maximum number of CD8^+^ T cells synthesizing TNF-α and IFN-γ was observed after 48 h, which was considerably reduced after 1 week, indicating that the capacity to produce cytokines was weakened after 1 week. With the recovery of the disease and the reduction in inflammation after 2 weeks, the expression of PD-1 and Tim-3 on the surface of CD8^+^ T cells was reduced, but the level was still higher than that in the control group, and the ability of these cells to synthesize cytokines also recovered (Fig. 2d, f, g).

When mice were intraperitoneally injected with 17-AAG one week after the LPS-induced ALI model was established, CD8^+^ T cells showed increased expression of negative costimulatory molecules (PD-1, Tim-3), and their capacity to secrete the cytokines TNF-α and IFN-γ was further diminished in comparison to those in the untreated group (Fig. 3b–d, f, g).

### Inhibition of HSP90α worsened the prognosis of ALI mice

Survival analysis of the untreated group and 17-AAG-treated ALI group suggested that inhibiting HSP90α expression decreased the survival rate of ALI mice, and the prognosis of mice in this group was worse (*P* < 0.05) (Fig. 1f).

### HSP90α/STAT1/TOX was involved in the phenotypic changes of CD8^+^ T cells in LPS-induced ALI

**Fig. 4:**
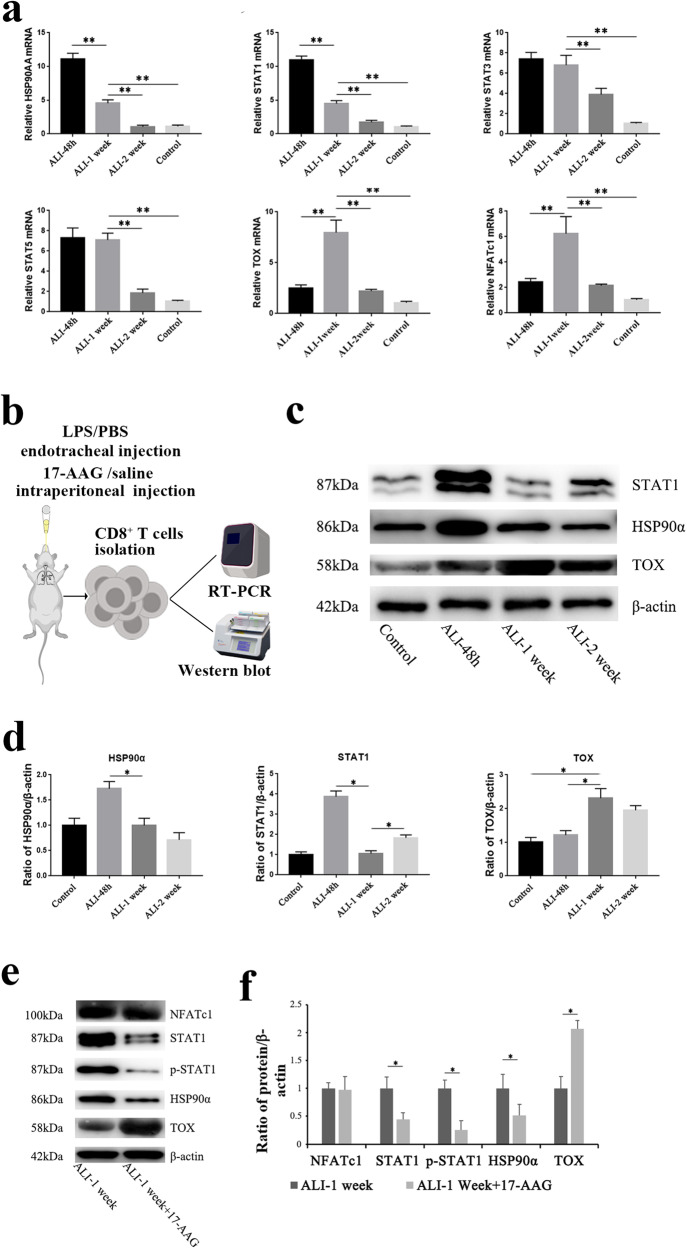
Changes in transcription factors in CD8^+^ T cells after ALI. **a** Expression of transcription factors associated with CD8^+^ T-cell exhaustion after ALI induction by LPS. **b** Schematic illustration of the experiments carried out on CD8^+^ T cells isolated from the ALI/17-AAG intervention groups. **c** Representative Western blot results of HSP90α, STAT1, and TOX in different ALI groups. **d** Quantitative analysis of HSP90α, STAT1, and TOX. **e** Representative Western blot results of NFATc1, HSP90α, p-STAT1, STAT1, and TOX in ALI-1 week and 17-AAG intervention groups. **f** Quantitative analysis of NFATc1, HSP90α, p-STAT1, STAT1, and TOX.

RT-PCR was used to analyze the mRNA expression of HSP90α, STAT1, STAT3, STAT5, TOX, and NFATc1 in the CD8^+^ T cells of mice at 48 h, 1 week and 2 weeks after LPS induction of ALI and the control group. The results are presented in Fig. 4a. HSP90α, STAT1, STAT3, and STAT5 mRNA peaked 48 h after ALI, but the exhaustion-related transcription factors TOX and NFATc1 were expressed at low levels. After 1 week of LPS-induced ALI, the expression of exhaustion-related transcription factors TOX and NFATc1 increased. However, STAT1 and HSP90α were considerably decreased compared to 48 h. STAT3 and STAT5 did not change significantly. Therefore, we hypothesized that HSP90α was involved in the regulation of CD8^+^ T-cell exhaustion through the regulation of STAT1.

### HSP90α and STAT1 inhibited CD8^+^ T-cell exhaustion

We further verified the HSP90α/STAT1/TOX signaling pathway by Western blotting. As shown in Fig. 4c and d, like mRNA expression, the protein expression of HSP90α and STAT1 peaked at 48 h, and TOX peaked one week later. However, the expression of HSP90α and STAT1 was downregulated at one week. It was discovered that the protein expression of HSP90α and STAT1 was negatively correlated with the expression of TOX, a crucial transcriptional regulator of CD8^+^ T-cell exhaustion, which was consistent with the analysis of negative costimulatory molecules and cytokines in earlier experiments [16]. As shown in Fig. 4e and f, inhibiting HSP90α with 17-AAG reduced STAT1 and increased TOX but did not affect NFATc1.

These findings suggested that the exhaustion phenotype of CD8^+^ T cells may be adversely linked with the expression of HSP90α and STAT1. Inhibiting HSP90α expression in ALI may increase the expression of exhaustion markers in CD8^+^ T cells and decrease their capacity for proliferation and cytokine release.

### Stimulating or inhibiting HSP90α induced phenotypic alterations of CD8^+^ T cells

**Fig. 5:**
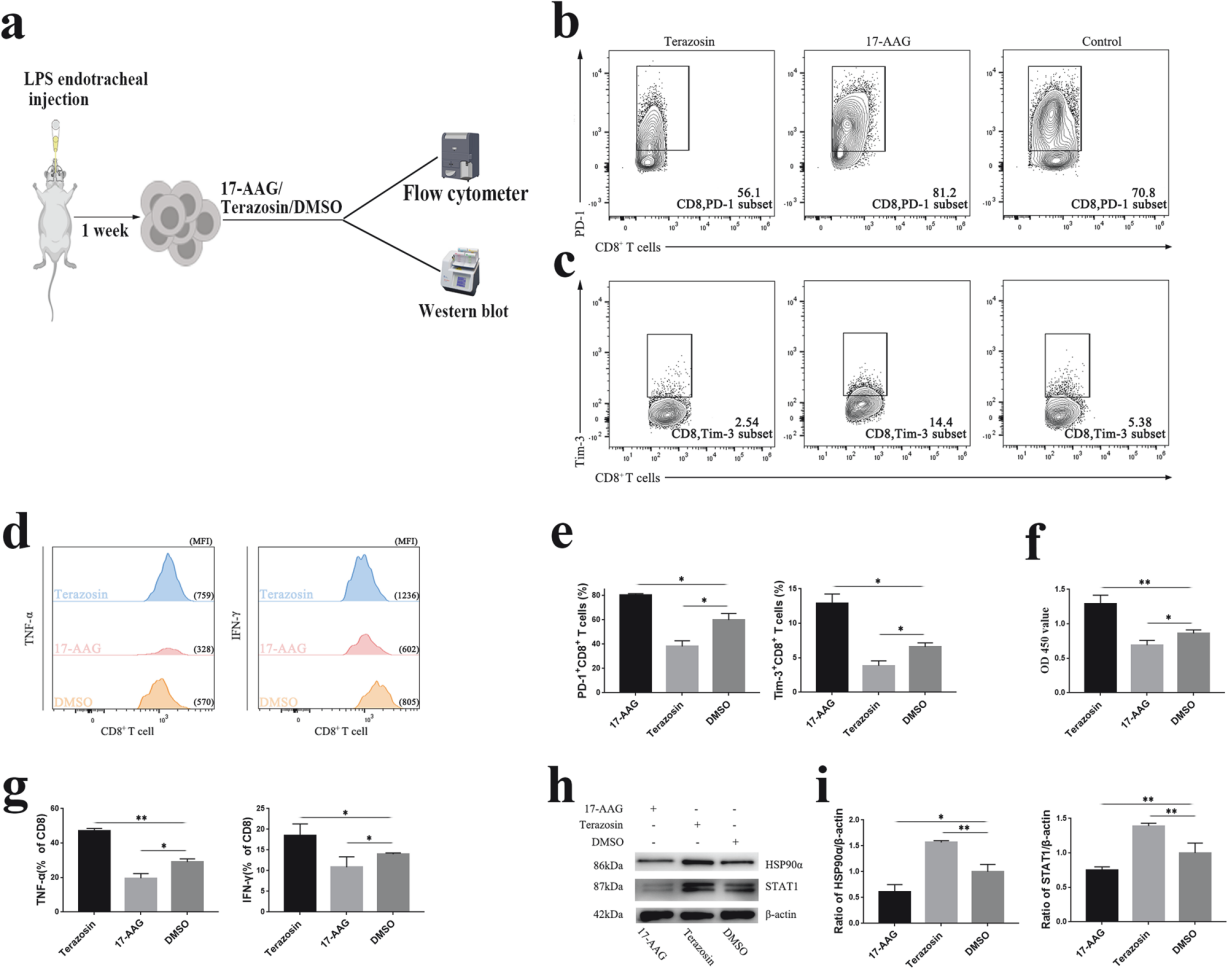
Effect of stimulation/inhibition of HSP90α on CD8^+^ T-cell exhaustion. **a** Schematic illustration of drug stimulation/inhibition of CD8^+^ T cells. **b** and **c** Representative flow plots showing PD-1 and Tim-3 in CD8^+^ T cells after the addition of Terazosin/17-AAG. **d** Representative flow charts of TNF-α and IFN-γ synthesis by CD8^+^ T cells after the addition of Terazosin/17-AAG. **e** Statistical analysis of PD-1 and Tim-3 in CD8^+^ T cells. **f** Comparison of the proliferation rate of CD8^+^ T cells after the stimulation/inhibition of HSP90α. **g** Statistical analysis of TNF-α and IFN-γ expressed by CD8^+^ T cells. **h** Typical bands of HSP90α and STAT1 after adding terazosin/17-AAG to CD8^+^ T cells. **i** Results of the quantitative analysis of HSP90α and STAT1.

In the previous study, the proliferation and function of CD8^+^ T cells in mice were the weakest after 1 week of ALI. To further verify the effect and regulatory mechanism of HSP90α, CD8^+^ T cells were isolated and cultured after 1 week of ALI modeling. After the addition of an HSP90α stimulator (terazosin) and suppressor (17-AAG), CD8^+^ T-cell proliferation and the expression of negative stimulatory molecules (PD-1, Tim-3) and cytokines (IFN-γ, TNF-α) were examined. As shown in Fig. 5b–g, after the HSP90α agonist was added to CD8^+^ T cells in vitro, cell proliferation was enhanced, the expression of PD-1 and Tim-3 was decreased, and the expression of cytokines (TNF-α and IFN-γ) was increased. In contrast, CD8^+^ T cells exposed to HSP90α inhibitors showed reduced viability, increased production of negative costimulatory markers, and reduced cytokine production. These results suggested that HSP90α may play a role in controlling CD8^+^ T-cell phenotypic transformation.

### Agonism or inhibition of HSP90α influenced the expression of STAT1

The exhaustion phenotype of CD8^+^ T cells was most obvious at 1 week after LPS-induced ALI, and the expression of HSP90α and STAT1 was significantly downregulated at this time. Therefore, we analyzed the changes in STAT1 expression in CD8^+^ T cells isolated from the ALI-1-week group. After adding HSP90α agonists/inhibitors in vitro, the Western blot results confirmed that the change in STAT1 was consistent with that in HSP90α (Fig. 5h and i). Based on these results, we hypothesized that HSP90α could regulate the downstream signaling of CD8^+^ T cells by regulating STAT1 expression.

### HSP90α and STAT1 synergistically orchestrated CD8^+^ T-cell exhaustion

**Fig. 6:**
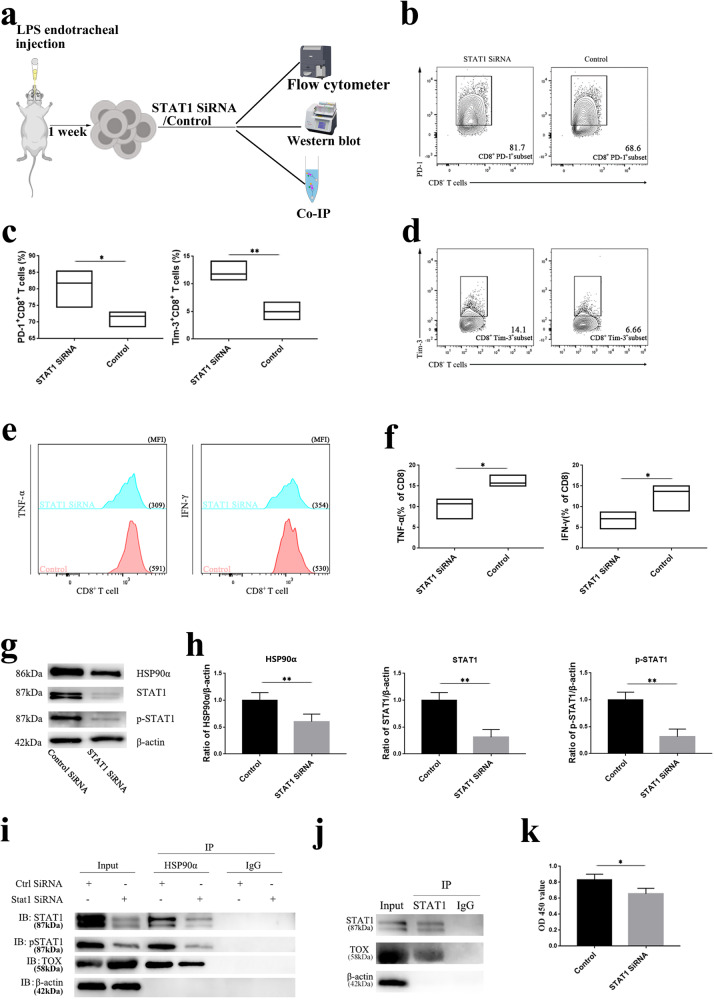
Effect of STAT1 interference on CD8^+^ T-cell exhaustion. **a** Schematic illustration of STAT1 interference in CD8^+^ T cells. **b** and **d** Representative flow plots showing PD-1 and Tim-3 expressed by CD8^+^ T cells after STAT1 interference. **c** Statistical analysis of PD-1 and Tim-3 expressed in CD8^+^ T cells. **e** Representative flow charts of TNF-α and IFN-γ synthesis by CD8^+^ T cells after STAT1 interference. **f** Statistical analysis of TNF-α and IFN-γ expressed by CD8^+^ T cells. **g** Representative bands showing HSP90α, STAT1, and p-STAT1 expression in control and STAT1 siRNA-transfected CD8^+^ T cells. **h** Statistical analysis of HSP90α, STAT1, and p-STAT1. **i** The interaction of CD8^+^ T cell HSP90α with STAT1 and TOX. **j** The interaction of STAT1 with TOX. **k** Comparison of the proliferation rate of CD8^+^ T cells after STAT1 interference.

To further verify the downstream regulatory mechanism of HSP90α, we next performed siRNA interference on the transcription factor STAT1. The Western blot results indicated that the expression of HSP90α was decreased after STAT1 interference, indicating that the expression of HSP90α was dependent on STAT1 (Fig. 6g, h). Furthermore, with the decrease in the expression of STAT1 and HSP90α, the proliferation of CD8^+^ T cells decreased (Fig. 6k), the expression of negative costimulatory molecules increased (Fig. 6b–d), and the ability of these cells to synthesize cytokines decreased (Fig. 6e and f). Therefore, these results suggested that HSP90α regulation of CD8^+^ T-cell exhaustion was dependent on STAT1 and that these two proteins play a synergistic role in CD8^+^ T-cell exhaustion.

### HSP90α modulated TOX directly or indirectly through STAT1

Finally, to further examine the proteins that interact with HSP90α in CD8^+^ T cells, we used Co-IP and Western blotting to precipitate HSP90α and detect proteins that interact with HSP90α. The expression of STAT1, p-STAT1, and TOX was observed in anti-HSP90α immunoprecipitation, demonstrating that HSP90α could bind to STAT1 and TOX in CD8^+^ T cells (Fig. 6i).

In CD8^+^ T cells STAT1 can also bind TOX directly (Fig. 6j). The expression of TOX was increased after STAT1 was disrupted, indicating that STAT1 was involved in the regulation of TOX in CD8^+^ T cells (Fig. 6i). HSP90α binding to TOX was decreased after STAT1 was knocked down. These results suggest that, in addition to the direct binding of HSP90α to TOX to govern the phenotypic transformation of CD8^+^ T cells, HSP90α may regulate TOX protein indirectly via STAT1.

## Discussion

Immune defense against intracellular infections depends on immune cells. In response to intense and persistent inflammation and antigenic signaling, lymphocytes always gradually differentiate into an exhausted phenotype [15, 17, 18]. In this study, the ALI model was constructed by injecting LPS into the trachea of mice. Pathological examination of lung tissue indicated that in the acute stage of LPS-induced lung injury, a large number of inflammatory cells accumulated in the lung tissue, and cytokines were significantly increased in serum and alveolar lavage fluid. However, with time and the self-repair of the immune system, immune cells gradually decreased in the lung tissue of surviving mice in the absence of any therapeutic intervention. On the one hand, it is possible that as the disease improves, the inflammation in lung tissue subsides, and immune cells are redistributed in vivo. On the other hand, the proliferation and function of immune cells may be weakened by the continuous stimulation of antigens/inflammation, leading to a decrease in the accumulation of inflammatory cells in the lung.

CD8^+^ T cells play an essential role in immune monitoring and the adaptive immune response against microbial infection [19]. When T-cell receptors are triggered by external antigens or pathogens, these cells quickly activate and proliferate. CD8^+^ T cells respond to antigens and pathogens by secreting immunoregulatory cytokines and chemokines or by direct killing [19].

Previous studies revealed that when pathogenic germs were not eliminated quickly, and antigenic and inflammatory stimuli remained, T cells developed an exhaustion phenotype [15, 20, 21]. Exhausted T cells are characterized by decreased proliferative capacity, reduced cytokine secretion, and increased expression of multiple inhibitory receptors, such as PD-1 and Tim-3, in response to sustained antigenic/inflammatory signals [22–24]. Therefore, in this study, we established a model of ALI induced by LPS and focused on whether the CD8^+^ T cells of ALI mice underwent exhaustion. The correlation between the expression of HSP90α and the proliferation and function of CD8^+^ T cells was also analyzed. We found that the proliferation and function of CD8^+^ T cells in mice after 1 week of ALI were the weakest, and PD-1 and Tim were highly expressed in these cells.

The expression of HSP90α increased in the acute stage of sepsis, and continuous high expression of HSP90α may promote the acute inflammatory stress response associated with sepsis [25]. In recent years, HSP90α and its copartners have also been discovered to be upregulated after T-cell activation. HSP90α has been demonstrated to be crucial in controlling several protein kinases that are required for T-cell activation [26]. Based on the GEO database, our earlier work found that patients with ARDS induced by sepsis had significantly lower levels of HSP90AA1 one week after admission than those with uncomplicated sepsis [7]. We also found a low number of CD8^+^ T cells and conversion to an exhaustion phenotype in sepsis-related ARDS patients [8]. So we postulated that HSP90α generated by HSP90AA1 was involved in the regulation of CD8^+^ T-cell phenotypic changes in sepsis-related ARDS.

In this research, one week after the establishment of the LPS-induced ALI model, untreated CD8^+^ T cells exhibited decreased proliferation, increased expression of the inhibitory molecules PD-1 and Tim-3, decreased cytokine secretion, and significantly increased expression of exhaustion transcription factors such as TOX. This finding suggested that CD8^+^ T cells were gradually exhausted after one week of continuous antigenic stimulation. Inhibiting HSP90α expression with 17-AAG further reduced the proliferation and proinflammatory activity of CD8^+^ T cells. Survival analysis of the untreated group and 17-AAG-treated ALI group suggested that inhibiting HSP90α expression decreased the survival rate of mice, and the prognosis of mice in this group was worse (*P* < 0.05). Therefore, we hypothesized that inhibiting HSP90α could impair lymphocyte activity and reduce their function in mediating inflammatory responses and killing pathogens, which adversely affected the prognosis of acute lung injury (Fig. 1f).

After further research, we found that the expression of HSP90α and STAT1 peaked at 48 h after ALI and decreased significantly by 1 week after ALI, suggesting that HSP90α and STAT1 may negatively regulate the exhaustion transformation of CD8^+^ T cells together. Inhibiting HSP90α expression with 17-AAG in LPS-induced ALI mice reduced STAT1 expression and increased TOX expression in CD8^+^ T cells but did not change NFATc1, suggesting that HSP90α regulates CD8^+^ T-cell exhaustion through a mechanism other than NR4A/NFATc1/TOX. This finding indicates that the control of CD8^+^ T-cell exhaustion in sepsis-associated ALI differs noticeably from the regulation of CD8^+^ T-cell exhaustion that is often triggered by tumors [27–29].

HSP90α is an important and abundant ATP-dependent molecular chaperone whose expression is induced by heat shock and other cellular stresses [10]. HSP90α plays an important role in cellular protein stabilization in eukaryotes by remodeling and activating client proteins such as signaling proteins, transcription factors, and regulatory kinases [30]. Tamspiramycin (17-AAG) is an effective HSP90α inhibitor that inhibits the chaperone action of HSP90α, causes the loss of function of HSP90α client proteins, and inhibits downstream signal transduction regulated by HSP90α [31]. On the other hand, terazosin is a widely marketed α1-adrenergic receptor antagonist that has been shown to enhance HSP90α activity by activating phosphoglycerate kinase 1 to accelerate the release of ATP [32]. In this study, after one week of acute lung injury, CD8^+^ T cells were isolated and cultured in vitro, and HSP90α expression was stimulated/ inhibited by medications. The results showed that activation or inhibition of HSP90α affected CD8^+^ T-cell proliferation, inflammatory effects, and negative costimulatory molecules on the cell surface.

The in vitro experiment confirmed that HSP90α could stimulate CD8^+^ T-cell proliferation, increase cytokine generation, and block the expression of negative stimulatory molecules (Fig. 5a–g). The transcription factor STAT1 was also increased or decreased with changes in HSP90α expression after pharmacological stimulation of CD8^+^ T cells (Fig. 5h and i). The in vitro findings suggested that HSP90α and STAT1 may have synergistic roles in the regulation of CD8^+^ T-cell exhaustion.

Several important transcription factors and regulatory proteins are required to differentiate T cells [33, 34]. Among them, TOX is an important transcription factor involved in maintaining the phenotype of exhausted T cells during chronic infection [35]. Previously, it was discovered that upregulating the negative costimulatory molecule PD-1 through TOX might alter the CD8^+^ T-cell exhaustion phenotype [28]. Removal of the DNA binding domain of TOX decreases PD-1 expression at the mRNA and protein levels, increases CD8^+^ T-cell cytokine production, and promotes the conversion of cells to effector T cells [16].

Previous studies have shown that TOX expression is susceptible to STAT1 regulation in patients with Sjogren’s syndrome [36]. STAT1 may also play an important role in regulating the expression of immune checkpoint molecules in exhausted T cells [37]. Stat1^−/−^ murine T cells had impaired synthesis and secretion of tumor necrosis factor, reduced proliferative activity, and upregulated expression of the inhibitory receptors PD-1 and CD69 [37, 38]. In this experiment, the in vitro results showed that the transcription factor STAT1 was necessary for HSP90α expression, and HSP90α could stabilize and activate STAT1. Therefore, we hypothesized that HSP90α was involved in the regulation of the CD8^+^ T-cell exhaustion phenotype via STAT1 regulation of the transcription factor TOX.

To verify the hypothesis, we further interfered with the expression of STAT1 in CD8^+^ T cells, and the expression of HSP90α was decreased after the expression of STAT1 was inhibited. Furthermore, after the decrease in the expression of STAT1 and HSP90α, CD8^+^ T cells had impaired proliferative viability, increased expression of negative costimulatory molecules, and decreased ability to synthesize cytokines. Thus, the regulatory role of HSP90α was dependent on the involvement of STAT1, and HSP90α could stabilize and activate the transcription factor STAT1. HSP90α and STAT1 may have synergistic roles in the regulation of CD8^+^ T-cell exhaustion [33].

Finally, co-IP was used to examine the interaction of HSP90α, STAT1, and TOX in CD8^+^ T cells. The presence of STAT1, p-STAT1, and TOX was detected in the anti-HSP90α immunoprecipitates, indicating that HSP90α could simultaneously bind to STAT1 and TOX in CD8^+^ T cells (Fig. 6i). When STAT1 expression decreased, the binding of TOX and HSP90α also decreased accordingly. However, it cannot be denied that the reduced STAT1 might lead to the downregulation of HSP90α, resulting in decreased binding to TOX. To confirm the findings, we performed a co-IP analysis of STAT1 and TOX, which revealed that STAT1 and TOX may bind directly to each other. Therefore, we conclude that in addition to direct binding to TOX, another component of HSP90α may indirectly affect the function of the TOX protein through STAT1 (Fig. 6i, j).

Our study included limitations that should be addressed. First, the ALI model used in this study, which was created by endotracheal infusion of LPS, cannot accurately depict real sepsis-related ARDS patients, particularly those with initial infection in nonlung tissues. Sepsis is the most prevalent cause of ARDS, and the pathogens involved include viruses, bacteria, and fungi. The LPS employed in this study is mostly from Gram-negative bacteria, hence the findings may not apply to all ARDS patients. Second, lung tissue and CD8^+^ T cells were collected at various periods following modeling from surviving animals, and based on past clinical investigations, it is probable that T-cell exhaustion was more severe in deceased mice. Finally, HSP90α is a molecular chaperone with more than 200 client proteins, and additional research is needed to determine if this factor controls changes in CD8^+^ T cells via other transcription factors and signaling pathways.

## Conclusions

**Fig. 7:**
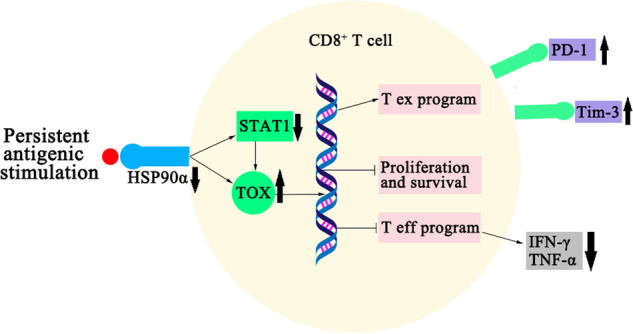
The regulatory mechanism of the HSP90α/STAT1/TOX pathway during CD8^+^ T-cell exhaustion. Schematic diagram showing the mechanism by which HSP90α regulates CD8^+^ T-cell exhaustion.

We discovered that HSP90α adversely influenced CD8^+^ T-cell exhaustion in sepsis-associated ALI in this study. HSP90α promoted the expression of STAT1, inhibited the expression of the exhaustion-related transcription factor TOX and negative stimulatory molecules PD-1 and Tim-3, promoted the proliferation of CD8^+^ T cells, and improved the secretion of inflammatory cytokines in both in vitro and in vivo models (Fig. 7). To regulate CD8^+^ T cells, HSP90α requires the synergistic engagement of STAT1. HSP90α can bind directly to the transcription factor TOX or indirectly regulate the TOX-mediated exhaustion signaling cascade via STAT1. According to our findings, the signaling axis involving HSP90α-STAT1-TOX has emerged as a potentially valuable target for monitoring CD8^+^ T-cell exhaustion. Identifying comparable therapeutic targets for HSP90α to modify the CD8^+^ T-cell phenotype and regulate the systemic inflammatory response in sepsis-associated ARDS patients is likely to bring new ideas for preventing and treating this disease.

## Materials and methods

### Mice and experimental design

Male C57BL/6J mice (8–9 weeks, 20–22 g, male) were purchased from the SLAC Laboratory (Shanghai, China). The animals were maintained in specific pathogen-free facilities and infected in modified pathogen-free facilities. All animal procedures conformed to the ARRIVE guidelines and were approved by the Animal Ethics Committee of Fudan University. Animals were assigned to five experimental groups (10 mice in each group): the control group, ALI 48-h group, ALI-1-week group, ALI-1-week + 17-AAG group, and ALI 2-week group. The mice were grouped by computer randomization. Animal managers and experimentalists do not know the distribution order; Researchers assessing, testing, or quantifying experimental results do not see the intervention. No mice were excluded from the experiment.

The sepsis-induced ALI model was established by intratracheal instillation of 15 mg/kg LPS (Sigma Chemical, Missouri, USA) [39]. An identical treatment was performed on control mice using intratracheal saline infusion. Blood, alveolar lavage fluid, lung tissue, and spleen tissue were collected for further testing at 48 h, Day 7, and Day 14 after ALI modeling in the ALI 48-hour group, 1-week group, and 2-week group, respectively. Mice in the ALI-1 week + 17-AAG group were intraperitoneally injected with 50 mg/kg 17-AAG at 24, 72, and 120 h after intratracheal instillation of LPS. On the 7th day, the mice were sacrificed, and their blood, alveolar lavage fluid, lungs, and spleen were collected for further testing (Fig. 1a).

### Bronchoalveolar lavage fluid (BALF) and blood analysis

BALF was obtained as previously described, and 1 mL PBS was intratracheally instilled into the right lung and then lavaged three times [40]. The removed fluid was immediately centrifuged (400 × *g*, 10 min). The supernatant was collected for further cytokine analysis. After sacrificing the mice, the blood was collected and centrifuged (400 × *g* for 10 min) immediately. The plasma was then used for cytokine analysis.

### Lung wet-to-dry weight ratio

The left upper lungs of each animal were excised and weighed instantly to assess the wet weight. To assess the dry weight, lung tissues were dehydrated in a drying oven at 50 °C until the dry weight of the lung was in a constant weight state. Then, the lung wet/dry ratio (*W*/*D*) was determined as follows: *W*/*D* = wet weight/dry weight × 100%.

### Survival studies

The survival rate of untreated mice and 17-AAG intervention mice was recorded every 6 h for 1 week after LPS intratracheal instillation. Each group contains 10 mice.

### Histopathologic evaluation

The left lower lungs were fixed in 10% formalin and then embedded in paraffin. For histopathologic analysis, the lesions (5 μm thick) were stained with hematoxylin and eosin. The images were captured by a light microscope (Olympus IX73, Tokyo, Japan). A five-point, semiquantitative, severity-based scoring system was used to assess the degree of alveolar hemorrhages, neutrophil infiltration, formation of hyaline membranes, alveolar wall thickness, and alveolar cavity fracture in each visual field graded as follows: 1 equals normal; 2 equals to 1–25% destruction of examined tissue; 3 equals to 26–50% destruction of examined tissue; 4 equals to 51–75% destruction of examined tissue; and 5 equals to 76–100% destruction of examined tissue.

### Immunofluorescence (IF) analysis

IF staining was used to show the changes in HSP90α and CD8^+^ T cells in the mouse lung tissue. After deparaffinization, the 5 μm thickness lung tissue (left) sections were permeabilized with 0.1% Triton X-100 and blocked with 5% bovine serum albumin (BSA) for 30 min. The sections were then incubated with HSP90α (Abcam, ab2928) and CD8 (Servicebio, GB114196) antibodies overnight at 4 °C. After washing, the sections were incubated with secondary antibodies for 1 h at room temperature. Cell nuclei were counter-stained with 4’6-diamidino-2-phenylindole (DAPI, Servicebio, G1012). Lastly, a fluorescence microscope (Olympus, Tokyo, Japan) was utilized to image tissue samples.

### Enzyme-linked immunosorbent assay (ELISA)

The concentration of inflammatory factors, TNF-α, IFN-γ and IL-2 in BALF and serum were detected using mouse ELISA kits (MultiSciences, 70-EK282/4-480; 70-EK280/3-480; 70-EK202/2-48), according to the manufacturer’s instructions. The optical density was measured using a microplate reader (Molecular, CA, USA) at wavelengths of 450 and 630 nm, and inflammatory factor concentrations were determined using a standard curve.

### Isolation and culture of spleen-derived CD8^+^ T cells

The mouse spleen was homogenized, and lymphocytes were extracted by density gradient centrifugation using Ficoll Paque Plus (Biosci, 7211011). According to the manufacturer’s instructions, CD8^+^ T cells were isolated by Mouse CD8a (Ly-2) MicroBeads (Miltenyi Biotec, 130-117-044). The purity of the CD8^+^ T cells isolated was >90% by a flow cytometric analysis. The CD8^+^ T cells were cultured in Roswell Park Memorial Institute 1640 medium supplemented with 10% fetal calf serum, 100 U/mL of penicillin, and 100 μg/mL of streptomycin at 37 °C under saturated humidity conditions and 5% CO_2_.

### In vitro stimulation and suppression assay

For ex vivo stimulation, 1 × 10^6^ CD8^+^ T cells were cultured in vitro for 24 h with 20 ng/mL recombinant mouse IL-2 (BioLegend, 575402) in 12-well plates, which were precoated with 5 μg/mL anti-mouse CD3 (BioLegend, 100239) and 0.5 μg/mL anti-mouse CD28 Abs (BioLegend, 302941). Then CD8^+^ T cells were incubated with 5 μM DMSO (Absin, abs9184) or 5 μM 17-AAG (Meilunbio, MB1634) or 5 μM Terazosin (Absin, abs816551) for 48 h.

### siRNA transfection

CD8^+^ T cells were cultured in vitro for 24 h with 20 ng/mL recombinant mouse IL-2 in 12-well plates, which were precoated with 5 μg/mL anti-mouse CD3 and 0.5 μg/mL anti-mouse CD28 Abs. For transfections, 2 × 10^6^ CD8^+^ T cells were transfected with a library of *stat11* siRNA with the following sequences: 5’ CUGUUACUUUCCCAGAUAUUATT 3’ and 5’ UAAUAUCUGGGAAAGUAACA GTT 3’. A scrambled siRNA was used as a control with the sequence: 5’ UUCUCCGAACGUGUCACGUTT 3’ and 5’ ACGUGACACGUUCGGAGAATT 3’. According to the manufacturer’s instructions, these siRNAs were transfected using Lipofectamine RNAiMAX (Thermo Fisher, L3000015). After transfection for 72 h, the cells were lysed for Western blots and Co-Immunoprecipitation. Anti-GAPDH was used to assess the transfection efficacy.

### Cell counting kit-8 (CCK8) assay

CD8^+^ T cells were purified as described previously and seeded into a 96-well plate. After being cultured for 24 h, 10 μL CCK8 reagent (Yeason, 40203ES60) was added to each well with incubation at 37 °C for 4 h. Then, the results were detected at 450 nm wavelength using a spectrophotometer (Molecular, CA, USA).

### Flow cytometry

To analyze surface markers, mouse CD8^+^ T cells or splenocytes were stained in PBS containing 5% FBS (FACS buffer) and antibodies targeting murine CD3 (FITC, BD Pharmingen, 553061), CD8a (PerCP-Cy5.5, BD Pharmingen, 551162), PD-1 (APC, BD Pharmingen, 562671) and Tim-3 (PE-CF594, BD Pharmingen, 566998)for 20 min at 4 °C, and washed once with FACS buffer.

To detect intracellular cytokines, cells were stimulated with Cell Activation Cocktail (with Brefeldin A) (BioLegend, 423303) for 6 h. After being stimulated, the cells were stained with antibodies targeting surface molecules and subjected to intracellular staining by using FIX and PERM kits (MultiSciences, 70-GAS003/2) according to the manufacturer’s instructions. Briefly, the cells were fixed and permeabilized, followed by the addition of antibodies targeting IFN-γ (PE, BioLegend, 163503)and TNF-α (APC, BioLegend, 506307). The cells were acquired on a flow cytometer (Beckton Dickinson, NJ, USA). The data were analyzed with FlowJo Version 10.2 software.

### RNA preparation and quantitative real-time PCR (qRT-PCR)

Total RNA was extracted from the CD8^+^ T cells using a Cell Total RNA Isolation Kit (Vazyme, RC112). cDNA was synthesized using HiScript Q RT SuperMix (Vazyme, R323). Quantitative RT-PCR was performed with SYBR Green qPCR Master Mix (Vazyme, Q711) using an ABI Prism 7500 real-time PCR system (Thermo Fisher). The mRNA expression level was calculated using the 2^−ΔΔCT^ method. GAPDH was used as an internal control, and the results for each sample were normalized to the housekeeping gene GAPDH. The primers used for each gene examined are shown in the supplementary material, Table S1.

### Western blot assay

CD8^+^ T cells were lysed on ice with Radio immunoprecipitation assay (RIPA) buffer (Beyotime, P0013C) containing protease inhibitor (Beyotime, P1005) and phosphatase inhibitor (Beyotime, P1050) for protein collection. After centrifugation at 14,000×*g* for 10 min at 4 °C, the supernatants were collected. Then, the protein concentration was quantified using the BCA Protein Quantitative Analysis Kit (Beyotime, P0009). Appropriate amounts of proteins were added to 7.5% sodium dodecyl sulfate–polyacrylamide gel electrophoresis followed by migration and transferred onto polyvinylidene difluoride membranes. After washing, the membranes were blocked with Quickblock™ blocking buffer (Beyotime, P0231) for 30 min and incubated with specific primary antibodies against β-actin (Abcam, ab8226), HSP90α (Abcam, ab2928), TOX (Abcam, ab155768), STAT1 (Abcam, ab239360), p-STAT1 (Abcam, ab109461) and NFATc1 (Abcam, ab2796) at 4 °C overnight. The following day, the membranes were washed in TBST and incubated with the corresponding second antibody for 1 h at room temperature. Immunoreactive bands were visualized by chemiluminescence using an ECL Kit (Beyotime, P0018FS). Protein quantification was performed using ImageJ software (https://imagej.net/).

### Co-immunoprecipitation (Co-IP)

CD8^+^ T cells were lyzed at 4 °C for 30 min in ice‐cold RIPA buffer. Supernatants were separated by centrifugation (14,000×*g* 10 min at 4 °C). Cell lysates were incubated with anti‐HSP90α (Abcam, ab2928), anti‐STAT1 (Abcam, ab239360), or the control IgG antibody (Beyotime, P2179M) for 16 h at 4 °C followed by a 2 h incubation with Protein A/G beads (Beyotime, P2179M) at 4 °C. After three washes with IP lysis buffer, protein samples were collected by boiling in 1 × SDS loading buffer for 10 min. Samples were separated using SDS–PAGE and further analyzed by performing immunoblotting. Immunoprecipitation with mouse IgG was taken as the negative control.

### Statistical analysis

Data were organized and analyzed using GraphPad Prism (version 7.0, CA, USA) and SPSS software (version 21, IL, USA). The Shapiro–Wilk test was used to check the normality and homogeneity of variance of all data. Differences between the two groups were analyzed by the two-tailed *t*-test. Multiple comparisons were performed by one-way analysis of variance (ANOVA) followed by the Bonferroni post hoc test, respectively. The survival curve was plotted using the Kaplan–Meier method. All data were expressed as mean ± standard error, and *p* < 0.05 was considered statistically significant. Each independent experiment was repeated at least 3 times.

### Supplementary information

The primer sequence of mRNA

Uncropped western blots

## Data Availability

Data described in this manuscript will be freely available upon request to any researcher wishing to use them for non-commercial purposes.

## Materials availability

Materials described in this manuscript will be freely available upon request to any researcher wishing to use them for non-commercial purposes.
