# Supplementary material for: Heat shock protein 90α reduces CD8+ T cell exhaustion in acute lung injury induced by lipopolysaccharide
Source: Cell Death Discov. 2024 Jun 13;10:283. doi: 10.1038/s41420-024-02046-8 (PMC11176380; doi:10.1038/s41420-024-02046-8)
Supplement: Supplementary file 1 — The primer sequence of mRNA [file 41420_2024_2046_MOESM1_ESM.docx]

**Table S1. The primer sequence of mRNA**

| Primer | Sequence |
| --- | --- |
| Gapdh Forward | 5’ CCCCTCTGGAAAGCTGTGGCGT 3’ |
| Gapdh Reverse | 5’ AGCTTCCCGTTCAGCTCTGG 3’ |
| NFATc Forward | 5’ CCTTAAGCCGCACGCCTTCTAC 3’ |
| NFATc Reverse | 5’ CCTTGGTGTTGCCTACGATCTTCTC 3’ |
| Tox Forward | 5’ GCACTGCTCTCCAATTCCATCTCTG 3’ |
| Tox Reverse | 5’ CTTGCCTGCTGTCTGATGTCTGTAG 3’ |
| Stat1 Forward | 5’ GCTGCCGAGAACATACCA 3’ |
| Stat1 Reverse | 5’ GGGTCGTCAAGCTCCATC 3’ |
| Hsp90aa Forward | 5’ GTGTGCAACAGCTGAAGGAA 3’ |
| Hsp90aa Reverse | 5’ CTCTCCATGTTTGCTGTCCA 3’ |
