# Supplementary figures and images for: Heat shock protein 90α reduces CD8+ T cell exhaustion in acute lung injury induced by lipopolysaccharide
Source: Cell Death Discov. 2024 Jun 13;10:283. doi: 10.1038/s41420-024-02046-8 (PMC11176380; doi:10.1038/s41420-024-02046-8)

**Figure 4c**

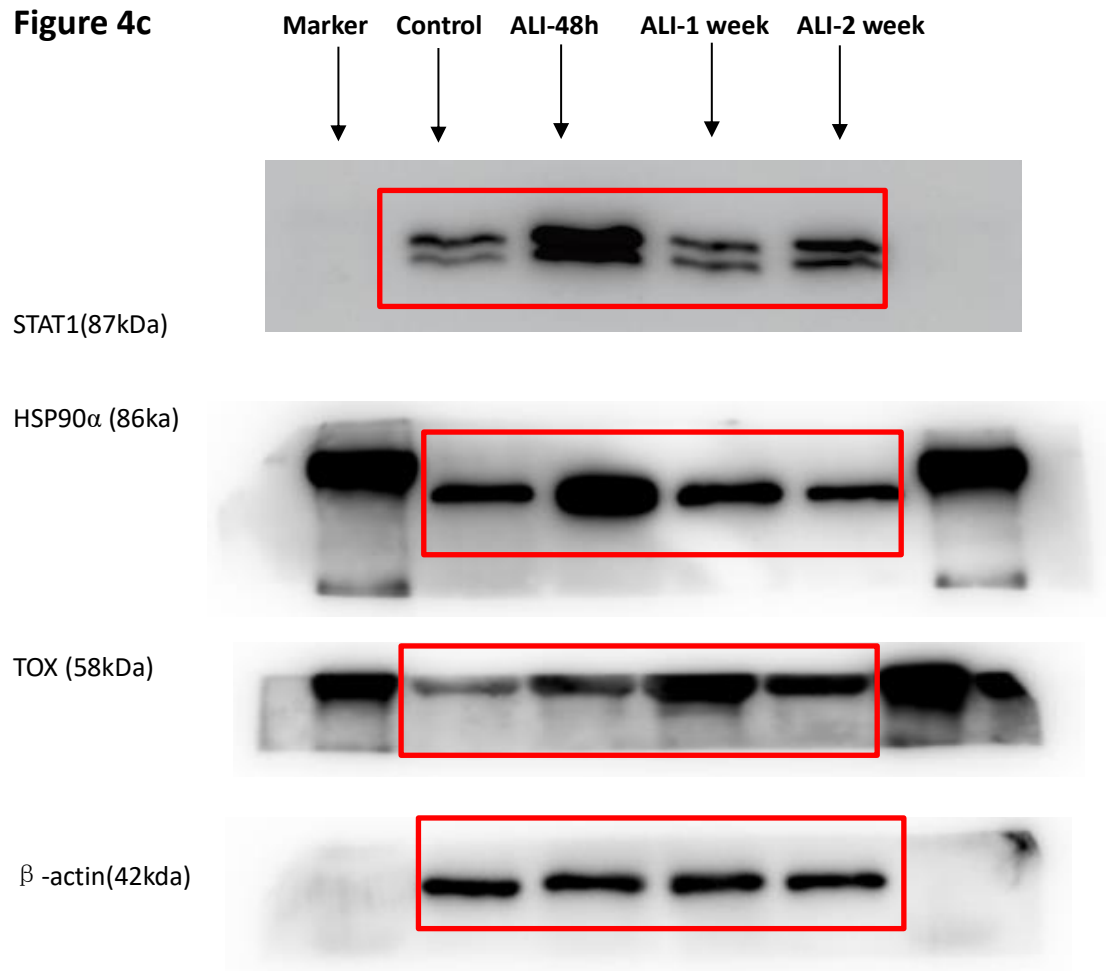

**Figure 4e**

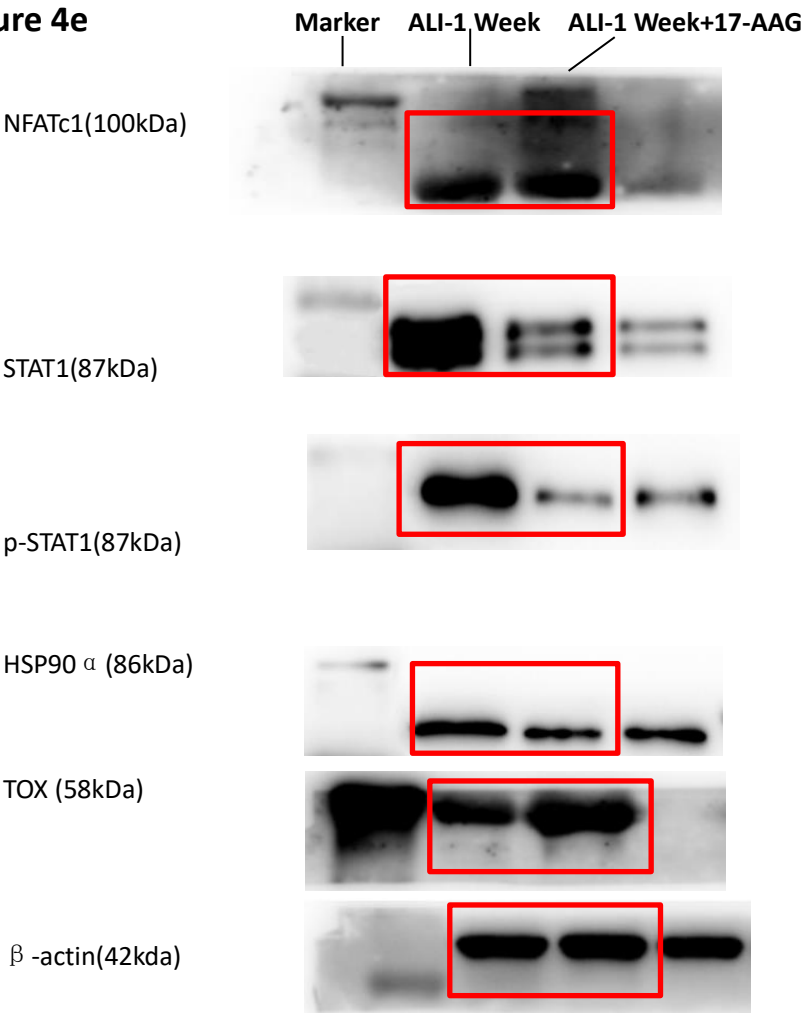

Figure 5h

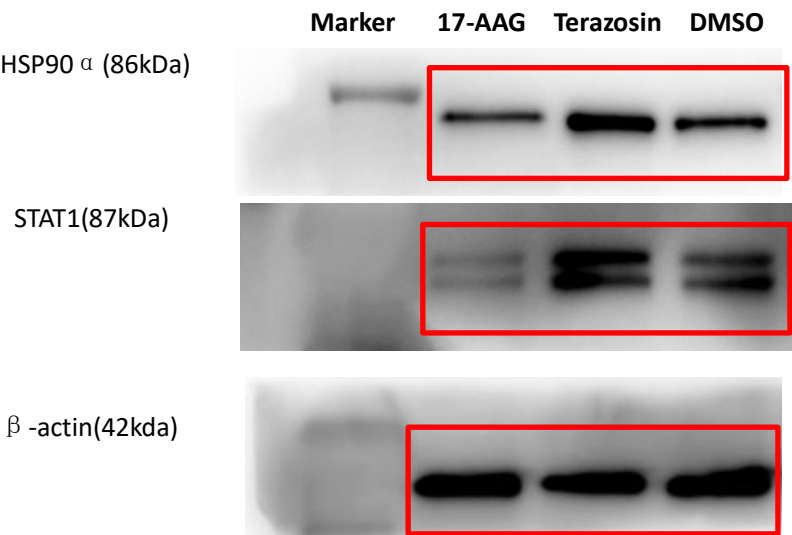

Figure 6g

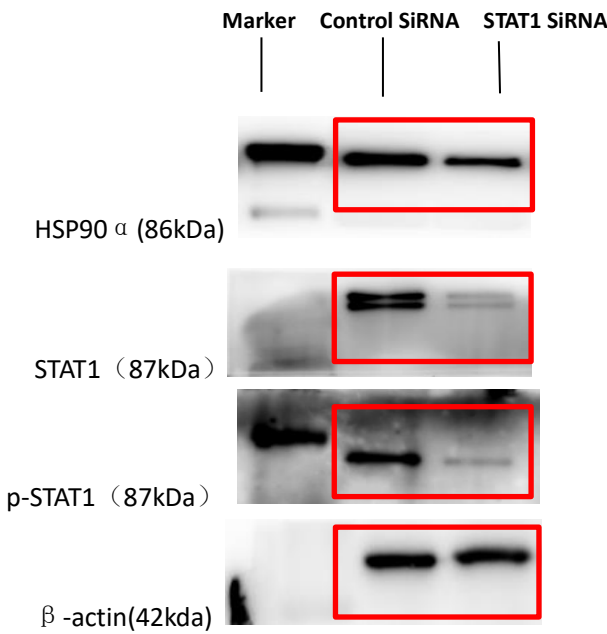

**Figure 6i**

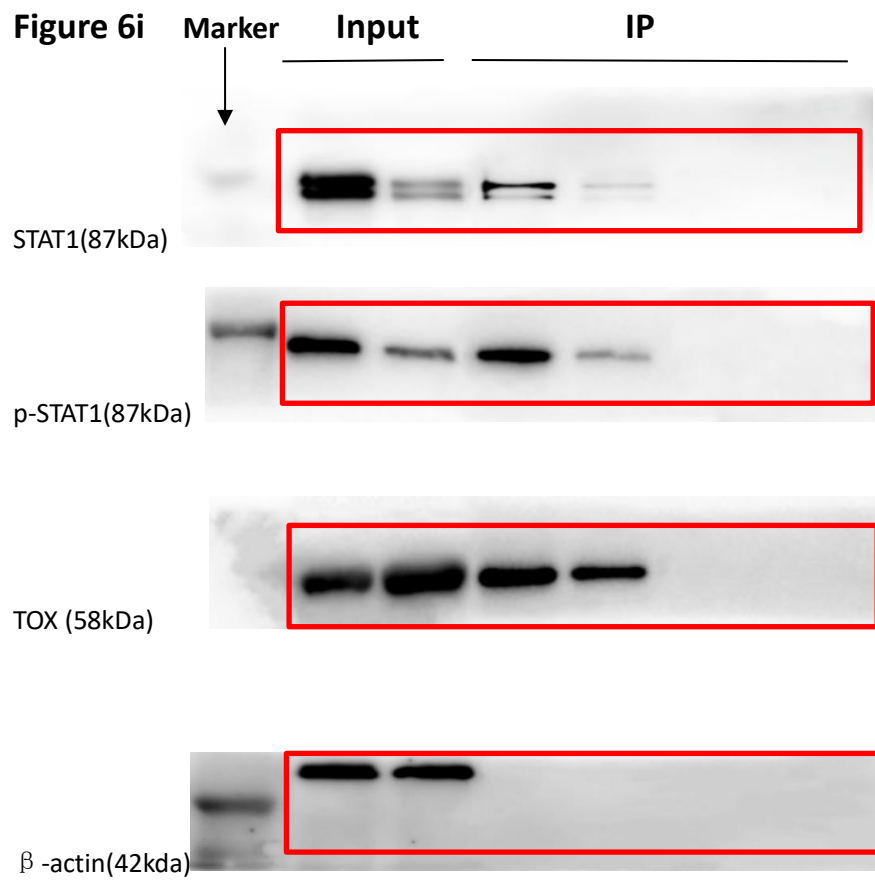

**Figure 6j**

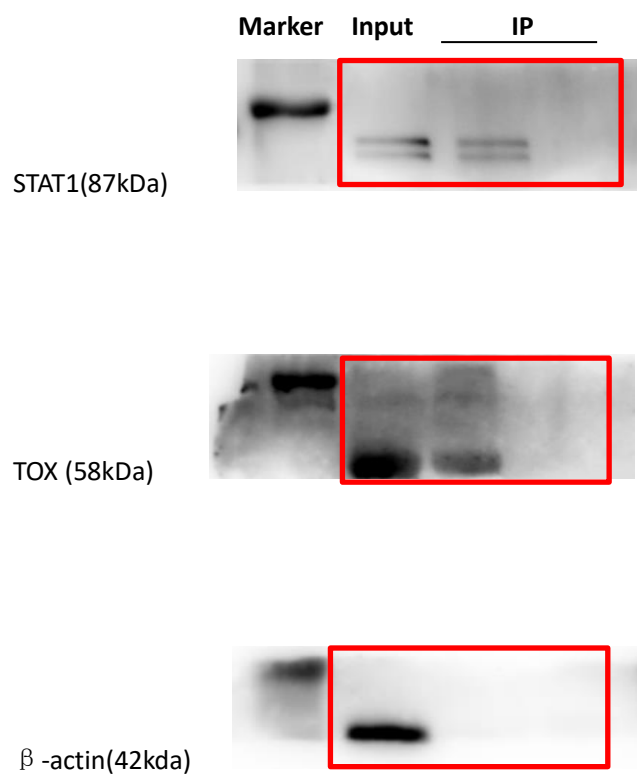

Supplement: Supplementary file 2 — Uncropped western blots [file 41420_2024_2046_MOESM2_ESM.pdf]
